# Gestational ethanol exposure impairs motor skills in female mice through dysregulated striatal dopamine and acetylcholine function

**DOI:** 10.1038/s41386-023-01594-4

**Published:** 2023-05-15

**Authors:** Sebastiano Bariselli, Yolanda Mateo, Noa Reuveni, David M. Lovinger

**Affiliations:** Laboratory for Integrative Neuroscience (LIN), NIH-NIAAA, 5625 Fishers Lane, Bethesda, MD 20892 USA

**Keywords:** Developmental disorders, Addiction

## Abstract

Fetal alcohol exposure has deleterious consequences on the motor skills of patients affected by Fetal Alcohol Spectrum Disorder (FASD) and in pre-clinical models of gestational ethanol exposure (GEE). Deficits in striatal cholinergic interneurons (CINs) and dopamine function impair action learning and execution, yet the effects of GEE on acetylcholine (ACh) and striatal dopamine release remain unexplored. Here, we report that alcohol exposure during the first ten postnatal days (GEE^P0-P10^), which mimics ethanol consumption during the last gestational trimester in humans, induces sex-specific anatomical and motor skill deficits in female mice during adulthood. Consistent with these behavioral impairments, we observed increased stimulus evoked-dopamine levels in the dorsolateral striatum (DLS) of GEE^P0-P10^ female, but not male, mice. Further experiments revealed sex-specific deficits in β2-containing nicotinic ACh receptor (nAChR)-modulation of electrically evoked dopamine release. Moreover, we found a reduced decay of ACh transients and a decreased excitability of striatal CINs in DLS of GEE^P0-P10^ females, indicating striatal CIN dysfunctions. Finally, the administration of varenicline, a β2-containing nAChR partial agonist, and chemogenetic-mediated increase in CIN activity improved motor performance in adult GEE^P0-P10^ females. Altogether, these data shed new light on GEE-induced striatal deficits and establish potential pharmacological and circuit-specific interventions to ameliorate motor symptoms of FASD.

## Introduction

Alcohol exposure during development leads to heterogeneous anatomical and neurobehavioral conditions, collectively known as Fetal Alcohol Spectrum Disorder (FASD) [1]. While a significant number of women with Alcohol Use Disorder (AUD) abstain during early pregnancy, a large proportion of them report alcohol use during perinatal periods [2, 3]. The teratogenic effects of ethanol include impaired cognitive flexibility and motor deficits [4, 5], severely affecting patient’s everyday life and resulting in high socioeconomic costs [6]. Despite a reduced overall survival rate and higher prevalence of FASD in males during infancy [7], the higher severity of dysmorphology and cognitive symptoms reported in females [8] points to sex-related differences in the neurobehavioral effects of FASD.

In animal models of gestational ethanol exposure (GEE), female offspring show decreased behavioral inhibition [9] and impaired extinction of aversive memories compared to males [10]. While GEE interferes with cognitive and motor skill learning [11–14], sex-specific effects on motor function remain unclear. Considering the high rate of alcohol relapse during perinatal periods [2] and the developmental similarities between the last trimester of human pregnancy and the first 10 postnatal days (P0-P10) in mice [15], we implemented a mouse model of binge-like and late-term gestational ethanol exposure that would cover this developmental period (GEE^P0-P10^).

We have previously hypothesized that the behavioral deficits induced by GEE might derive from synaptic and circuit deficits in the striatum [16], a brain region involved in cognitive and motor function [17–19] and particularly vulnerable to the teratogenic effects of fetal alcohol exposure [13, 20]. Dorsal striatal circuits contain different neuronal populations, including medium spiny neurons (MSNs), the primary striatal projection neurons, acetylcholine (ACh)-releasing interneurons (CINs), as well as dopaminergic inputs from the midbrain. Recent simultaneous in vivo fiber photometry experiments revealed coordinated ACh and dopamine signaling during decision-making [21] and spontaneous locomotion [22, 23] in the dorsal striatum. These data support previous evidence that ACh dysregulation impairs cognitive function and motor skills [24–26], the latter typically assessed with rotarod tasks in rodents. Moreover, Fast Scan Cyclic Voltammetry (FSCV) experiments demonstrate that pre-synaptic nAChR and glutamatergic receptor activation modulates electrically evoked or ACh-driven striatal dopamine release [27–30]. While previous experiments show that GEE impairs dopamine receptor expression and binding in rodents [31, 32] and monkeys [33], whether GEE alters striatal dopamine dynamics remains an open question.

In addition to striatal dopamine dysregulation, GEE impairs ACh function in several brain regions. In rats, GEE alters the developmental expression of choline acetyltransferase (ChAT) [34], the enzyme that catalyzes ACh synthesis, with no obvious changes in the expression of acetylcholinesterase (AChE), the enzyme that catalyzes ACh degradation, in nucleus basalis of Meynert (NBM), neocortex, or striatum [35, 36]. Along with changes in ACh metabolism, GEE decreases the number of ACh-releasing neurons in the basal forebrain, striatal regions [37], and NBM [35] in rats and mice. Moreover, GEE alters muscarinic receptor (mAChR) signaling in striatal and hippocampal regions [36, 38], the latter associated with an impaired K^+^/Ca^2+^-stimulated ACh release [39] in rats. Finally, clinical studies demonstrated profound changes in ACh metabolism in the telencephalon and cerebellum of patients affected by FASD [40]. However, whether GEE impairs striatal ACh dynamics to alter dopamine release and motor function remains unknown.

Here, we generated an animal model of late-term gestational ethanol exposure (GEE^P0-P10^) that reveals a higher vulnerability of female offspring to high alcohol levels. We also provide evidence of impaired motor skills in GEE^P0-P10^ females during adulthood. FSCV experiments indicate that increased evoked dopamine responses in DLS of GEE^P0-P10^ female mice accompany these behavioral deficits. Pharmacological experiments further show impaired nAChR-mediated modulation of electrically evoked-dopamine release in DLS of GEE^P0-P10^ mice. The β2-subunit specific nAChR antagonist dihydro-β-erythroidine hydrobromide (DhβE) induces a larger decrease of dopamine transients evoked by single-pulse electrical stimulation in GEE^P0-P10^ compared to CE^P0-P10^ (control exposure) female mice, pointing to an increased nAChR function in driving dopamine release. Ex vivo slice photometry experiments with the genetically encoded fluorescent ACh sensor GACh_3.0_ [41] also revealed a GEE-induced decrease in the decay of evoked ACh release in DLS, suggesting decreased striatal ACh levels. Finally, electrophysiological recordings in DLS slices of GEE^P0-P10^ females show reduced excitability of CINs. Pharmacological modulation of nAChRs and chemogenetic-mediated increase in CIN firing ameliorate motor behavior deficits in GEE^P0-P10^ female offspring. Altogether, our data provide novel evidence for a sex-dependent alteration of striatal ACh and dopamine function in a mouse model of FASD.

## Material and methods

### Experimental subjects

Pregnant WT C57BL/6J mice were purchased at E7 from the Jackson laboratory and used in the present study. For the chemogenetic experiments, we used Chat-ires-Cre mice (B6.129S-Chattm1(cre)Lowl/MwarJ; Jackson laboratory). Their progeny were used for ethanol (EtOH) vapor exposure, circuit, and behavioral assays. Experimental subjects were used in accordance with the *NIH Guide for the Care and Use of Laboratory Animals*. The experiments performed in this study were approved in the LIN-DL-1 protocol for animal use authorization by the Animal Care and Use Committee of the NIAAA Division of Intramural Clinical and Biological Research.

### Procedure for postnatal ethanol exposure and BAC measurements

Upon birth, pups (post-natal day 0, P0) and dams were exposed to air (control, CE) or EtOH vapor (Gestational Ethanol Exposure, GEE) by placing the animal’s home cages in air-tight plexiglass chambers. 190-proof EtOH was vaporized at a rate of 8–9 liter of air per minute and adjusted to reach an EtOH concentration of 0.100–0.1500 mg/dL in the vapor chambers [42]. Mice were exposed to EtOH vapor in a 16 h-ON/8 h-OFF pattern, typically from 5–7 pm to 9–11 am, for 7 times over 10 days with a 3-day in-between break. Specifically, each animal received 7 GEE exposures distributed over days P0-P3 (3 overnight exposures, 16-hr each) and P6-P10 (4 overnight exposures, 16-hr each). Blood Alcohol Concentration (BAC) was measured from trunk blood in pups between P3-P9. Mice were decapitated, and blood was collected through a glass capillary. Serum was obtained, diluted, and alcohol concentration was measured using a colorimetric assay (Pointe Alcohol Reagent Test) [43].

### Pup retrieval assay

Pup retrieval assays were conducted to assess maternal behavior [44]. At the end of the 16-hr-ON exposure to EtOH, dams and pups were removed from the vapor chamber. Dams with pups and their nest were moved to a clean home-cage-like arena for at least 5 min for habituation. A single pup-retrieval trial began upon removing one pup from the nest and its placement in the opposite corner of the home cage-like arena. The time to retrieve the pup was measured from the removal of the pup from the nest by the experimenter until it was placed back in the nest by the dam. Each trial lasted for a maximum of 120 seconds, and the session was concluded upon 10 consecutive trials with no breaks between trials.

### Animal surgeries

Animals aged 6–8 weeks were anesthetized in an induction chamber with 5% isoflurane. Upon induction of deep anesthesia and loss of toe-pinch reflex, animals were mounted on a stereotaxic frame that delivered isoflurane at 1–3% through a conical facemask for the whole duration of the surgery. The incision site was shaved and disinfected with an iodine-povidone solution. A scalpel blade was utilized to cut the skin and expose the skull. Small craniotomies of about 0.5 mm in diameter were performed using an electric drill. A Hamilton syringe pre-loaded with a viral solution was inserted in the brain parenchyma to target the dorsolateral striatum (DLS) at the following coordinates: AP: 0.0 mm, ML: ± 2.4 mm, DV: −3.4 mm from bregma. An injection volume of 500 nL was delivered at a rate of 75 nL per minute in each hemisphere. The syringe needle remained in the brain for a total duration of 10 minutes. Upon delivery of the viral solution, the needle was removed, the skull surface was disinfected with an iodine-povidone solution, and the skin wound was closed using skin glue. Animals received an injection of ketoprofen and were placed on a heating pad. Animals were moved back to the colony room, and their well-being was monitored for two days after the surgeries.

### Viruses and reagents

AAV9-hSyn-ACh4.3 was obtained from WZ-Bioscience (titer 4.6 × 10^13^ VG/mL). AAV8-DIO-hSyn-mCherry (titer 2.2 × 10^13^ GC/mL) and AAV8-DIO-hM3Dq-mCherry (titer 1.8 × 10^13^ GC/mL) was obtained from Addgene.

### Rotarod

CE^P0-P10^ and GEE^P10-P10^ experimental subjects were brought into the behavioral room and habituated to the environment for at least 30 minutes. Afterward, animals were positioned on an accelerating rotarod (AccuRotor EzRod, Omnitech Electronics, Inc.) at a constant speed of 4 rpm. Upon placement, the rotarod accelerated from 4 to 40 rpm over a 5-minute trial. A trial ended when the experimental subject dropped from the rod. A total of 5 trials with an inter-trial interval of at least 5 minutes were administered daily. The animals underwent a total of 4 sessions over 4 consecutive days. The rotating rod and the bottom of the arena were cleaned at the end of each session. We performed rotarod experiments in CE^P0-P10^ and GEE^P10-P10^ naïve, or varenicline and saline-treated animals at 8 weeks of age. For animals expressing either mCherry or hM3Dq, we allowed at least 6 weeks for viral expression and started rotarod training during weeks 12−14. Recent reports show that in C57BL6/J mice, rotarod performance remains stable between weeks 5-24 [45] and sharply declines at month 12 (week 48) [46]. On the day of the experiments, we i.p. injected Clozapine-N-Oxide (CNO) at a 5 mg/Kg dose 60 minutes before starting rotarod training. Another cohort of animals received i.p. injections of either saline or varenicline (Varenicline tartrate, Tocris, 1 mg/Kg) 30 minutes before starting rotarod training.

### Intraperitoneal injections

Varenicline tartrate (1 mg/Kg; Tocris, 3754) and Clozapine-N-Oxide (CNO) Dihydrochloride (5 mg/Kg; Tocris, 6329) were dissolved in NaCl 0.9% (saline) and injected 30 and 60 minutes before rotarod test, respectively.

### Brain Slice Fast-scan cyclic voltammetry recordings (FSCV)

We conducted FSCV experiments on adult mice (12−32 weeks old). Animals were anesthetized with isoflurane, the brains were removed, and 300 µm-thick coronal sections through the striatum were prepared (Leica VT1200S, Leica Biosystems, IL) in ice-cold carbogen-saturated (95% O_2_/5% CO_2_) cutting solution (in mM: Sucrose 194, NaCl 30, KCl 4.5, MgCl_2_ 1, NaHCO_3_ 26, NaH_2_PO_4_ 1.2, Glucose 10). Slices were then transferred to a chamber filled with oxygenated artificial cerebrospinal fluid (aCSF) (pH 7.4) containing (in mM): NaCl (126), KCl (2.5), NaHCO_3_ (25), NaH_2_PO_4_ (1.2), dextrose (10), HEPES (20), CaCl_2_ (2.4), MgCl_2_ (1.2), and L-ascorbic acid (0.4) kept at 32 °C and allowed to recover for 1 h until used for recordings. After the equilibration period, brain slices were transferred to the recording chamber and perfused at a rate of ~1.5 mL/minute with aCSF. Once the brain slice was in place, a bipolar stainless-steel stimulating electrode (Plastics One, Roanoke, VA) was placed in the region of interest, and a carbon fiber electrode was placed ~200 µm from the stimulating electrode. Cylindrical carbon fibers (T650 carbon fiber, 7 μm diameter, 150−175 μm exposed length; Goodfellow, PA) were inserted into a glass pipette. The carbon-fiber electrode was held at −0.4 V, and the potential was increased to 1.2 V and back at 400 V/second every 100 ms using a triangle waveform. Dopamine release was evoked by rectangular, electrical pulse stimulation (50−800 µA; 1 ms, monophasic) applied every 3−5 minutes with a NL 800 A Current Stimulus Isolator (Digitimer, Hertfordshire, UK) in a random, non-sequential order. Data collection and analysis were performed using the Demon Voltammetry and Analysis software suite. Carbon fiber electrodes were calibrated using 1.0 μM DA after recordings. Input-output (I/O) curves were generated to compare the sensitivity of evoked dopamine release across varying electrical stimulation intensities (50−800 µA, 1 ms). For pharmacological experiments, baseline responses were collected for 15−20 minutes before drugs (dissolved in aCSF) were bath applied as indicated for each experiment.

### Brain slice photometry recordings

Photometric recordings were conducted as previously described [47] on adult animals (12−32 weeks old). Mice were anesthetized with isoflurane, rapidly decapitated, brains extracted, and 300 µm thick coronal sections were prepared using a Leica vibratome (Leica VT 1200 S). The slices were hemisected and examined to ensure viral expression of GACh_3.0_ in the region of interest using an epifluorescent Zeiss AxioZoom microscope equipped with a GFP filter set. Slices were incubated at 32 °C for 30 minutes before being moved to room temperature for one hour before beginning the experiments. Brain slices with GACh_3.0_ expression were transferred to an upright Zeiss AxioSkop2 microscope mounted on an XY translational stage and equipped with a GFP filter set. Oxygenated aCSF (same composition as for FSCV recordings) was perfused at 1−1.5 mL/minute and warmed to 30−32 °C. The recording region of interest was located under 4X magnification, and a stainless steel twisted bipolar stimulating electrode (Plastics One, Roanoke, VA) was placed on the tissue surface near the area of GACh_3.0_ expression. Slices were visualized with a 40X objective (0.8 NA), and the field of view (~180 mm x 180 mm) was adjusted, so the stimulating electrode was just outside the field of view (~200 µm). Under 40X magnification, the focus was adjusted to a focal layer beneath the slice surface where fluorescent cells could be identified. Fluorescent transients were quantified with a PMT-based system (PTI D-104 photometer) coupled with a Digidata 1322 A (Molecular Devices LLC) to digitize the PMT signal (100−1000 Hz). Clampex and Clampfit software (Molecular Devices) were used to collect and analyze photometry data. A mechanical shutter (Uniblitz V25) was used to limit exposure to fluorophore-exciting light to discrete periods and minimize photobleaching of GACh_3.0_ between recordings.

### Brain slice electrophysiology

Slice physiology experiments were conducted on 250 µm thick coronal slices containing the dorsolateral striatum (DLS) of adult animals (12−32 weeks old). CE^P0-P10^ and GEE^P10-P10^ experimental subjects were anesthetized with a mixture of isoflurane/O_2_ and decapitated. Brains were sliced using a cutting solution containing: 4.5 mM KCl, 1.2 mM NaH_2_PO_4_, 10 mM Glucose, 26 mM NaHCO_3_, 194 mM sucrose, 124 mM NaCl and 1 mM MgCl_2_. Brain slices were incubated in artificial cerebrospinal fluid (aCSF) containing: 4.5 mM KCl, 1.2 mM NaH_2_PO_4_, 10 mM Glucose, 26 mM NaHCO_3_, 14.6 mM Sucrose, 124 mM NaCl, 1 mM MgCl_2_ and 2 mM CaCl_2_ at 28° for 40 minutes. Whole-cell electrophysiological recordings were conducted at 30–32 °C in ACSF submerged slices. The recording pipette contained the following internal solution: 140 mM K-Glu, 10 mM HEPES, 0.1 mM CaCl_2_, 2 mM MgCl_2_, 1 mM EGTA, 2 mM ATP-Mg, 0.2 mM GTP-Na. We identified putative cholinergic interneurons of the DLS according to their large cell soma, depolarized resting membrane potential, and presence of an Ih (hyperpolarization-activated cyclic nucleotide-gated, HCN) current. The Ih current was measured in voltage-clamp configuration by clamping CINs at −50 mV and injecting a negative voltage step of −50 mV [48]. Traces were not corrected. Pipette resistance was between 3 and 5 MΩ while access resistance (10–30 MΩ) was monitored in voltage-clamp configuration by a hyperpolarizing step of −10 mV. Data were excluded when the access resistance changed > 20%. We measured spontaneous activity as the average action potential frequency recorded for the first 2 minutes after entering the current-clamp configuration. Subsequently, excitability experiments were conducted by injecting incremental current steps of + 10 pA every 30 s. During excitability experiments, the membrane potential (RMP, mV) was determined as the average voltage measured before (I = 0) each positive step current injection. We conducted cell-attached experiments for hM3Dq validation in I = 0 mode with NaCl 0.9% as the intra-pipette solution. After entering the cell-attached configuration, we waited 5 minutes before recording baseline firing, and CNO was applied for 10−15 min. In each experiment, representative example traces were chosen as single responses from CINs. Electrophysiological responses were collected with a Multiclamp 700B-amplifier (Axon Instruments, Foster City, CA), filtered at 2.2 kHz, digitized at 5 Hz, and analyzed online and offline using pClamp and Clampex software (Molecular Devices).

### Statistical analysis

No power analysis was performed to pre-determine the sample size of animals, neurons, or slices. However, we included sample groups of similar size compared to similar studies from the literature. Outlier analysis on rotarod data was performed by excluding animals whose performance differed more than 1.7 standard deviations (SD) from the sample mean in either the first training trial or average performance. Based on this criterion, we excluded: 1 CE^P0-P10^ female, 2 GEE^P0-P10^ females, 1 GEE^P0-P10^ male, 2 CE^P0-P10^ saline-treated females, 2 GEE^P0-P10^ varenicline-treated females, 1 mCherry CE^P0-P10^ female, 2 mCherry GEE^P0-P10^ females, 1 hM3Dq CE^P0-P10^ female and 1 hM3Dq GEE^P0-P10^ female. The normality of the sample distribution was assessed with the Shapiro-Wilk test. We compared two-sample distributions using a two-tailed t-test or the Mann-Whitney test. Analysis of variance was conducted using one-way, two-way, and repeated-measures two-way ANOVA or mixed models and reported in each figure legend. P denotes the main effects, while # denotes the interaction between factors in the ANOVA analysis. Statistical significance was determined when *p* < 0.05. *Post-hoc* comparisons were conducted as appropriate and reported in each figure legend. Fast Scan Cyclic voltammetry and ex vivo photometry experiments were performed with the experimenter blinded to group affiliation. Statistical analysis and graphs were performed with GraphPad/Prism.

## Results

### Late-term GEE impairs maternal behavior and overall female offspring growth

**Fig. 1:**
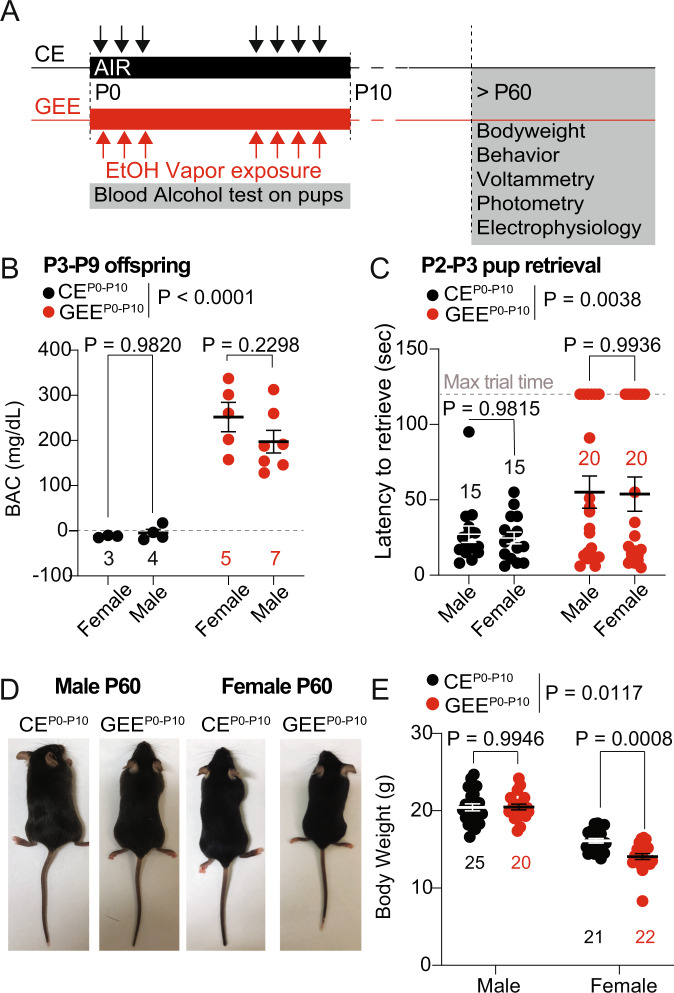
Late-term GEE impairs maternal behavior and overall offspring growth. **A** Timeline of ethanol vapor exposure. **B** Blood Alcohol Concentration (BAC) measured from male and female pups between P3-P9 (two-way ANOVA; treatment main effect: F_(1,15)_ = 72.64, *p* < 0.0001; sex main effect F_(1,15)_ = 0.7393, *p* = 0.4034; treatment × sex interaction: F_(1,15)_ = 1.282, *p* = 0.2752; followed by Sidak post-hoc test). **C** Pup retrieval latency for male and female pups between P2-P3 (two-way ANOVA; treatment main effect: F_(1,66)_ = 9.008, *p* = 0.0038; sex main effect F_(1,66)_ = 0.0383, *p* = 0.8455; treatment × sex interaction: F_(1,66)_ = 0.0041, *p* = 0.9491; followed by Sidak post-hoc test). The dotted line represents the maximum duration of each pup retrieval trial (120 sec, labeled Max trial time), and the red bar at 120 seconds shows the accumulated number of trials (13 in total) in which the GEE^P0-P10^ dams failed to retrieve their pups. **D** Representative image of adult CE^P0-P10^ and GEE^P0-P10^ male and female offspring. **E** Body weight of CE^P0-P10^ and GEE^P0-P10^ males and females at P60 (two-way ANOVA; treatment main effect: F_(1,84)_ = 6.640, *p* = 0.0117; sex main effect: F_(1,84)_ = 193.2, *p* < 0.0001; treatment × sex interaction: F_(1,84)_ = 7.323, *p* = 0.0082; Sidak post-hoc tests). Data are expressed as mean ± SEM. N indicates number of mice.

To model late-term gestational ethanol exposure (GEE^P0-P10^), C57BL/6J (Jackson Laboratory) dams and pups were placed in plexiglass chambers filled with vaporized ethanol (GEE^P0-P10^) or air (control exposure, CE^P0-P10^) [13] from postnatal day 0 (P0) to postnatal day 10 (P10). Pups and dams received seven total exposures of ethanol vapor in a 16 h-ON/8 h-OFF pattern with a 3-day break in between (Fig. 1A). Blood alcohol concentration (BAC) measurements between P3-P9 showed binge-like ethanol levels in the fetal circulation of both female and male pups (Fig. 1B). Thus, the intermittent exposure and the high ethanol levels in pups mimic binge-like cyclic patterns of alcohol consumption observed in women with alcohol use disorder. Given the negative impact of high-alcohol exposure on maternal care [49, 50], we performed pup retrieval tests on dams with CE^P0-P10^ and GEE^P0-P10^ litters at post-parturition day P2-P3. We observed that GEE^P0-P10^ dams needed more time to move their pups back to the nest compared to CE^P0-P10^, which is indicative of maternal neglect, independent of the sex of the pups (Fig. 1C). At P60, female GEE^P0-P10^ had lower body weight compared to CE^P0-P10^ females, with no detectable deficits in the male offspring (Fig. 1D, E). Altogether, our data reveal a sex-specific vulnerability of the female offspring to fetal alcohol exposure, which is not due to sex-related differences in ethanol levels but might derive from the combined teratogenic effects of ethanol and impaired maternal care.

### Late-term GEE impairs rotarod performance in the female offspring

**Fig. 2:**
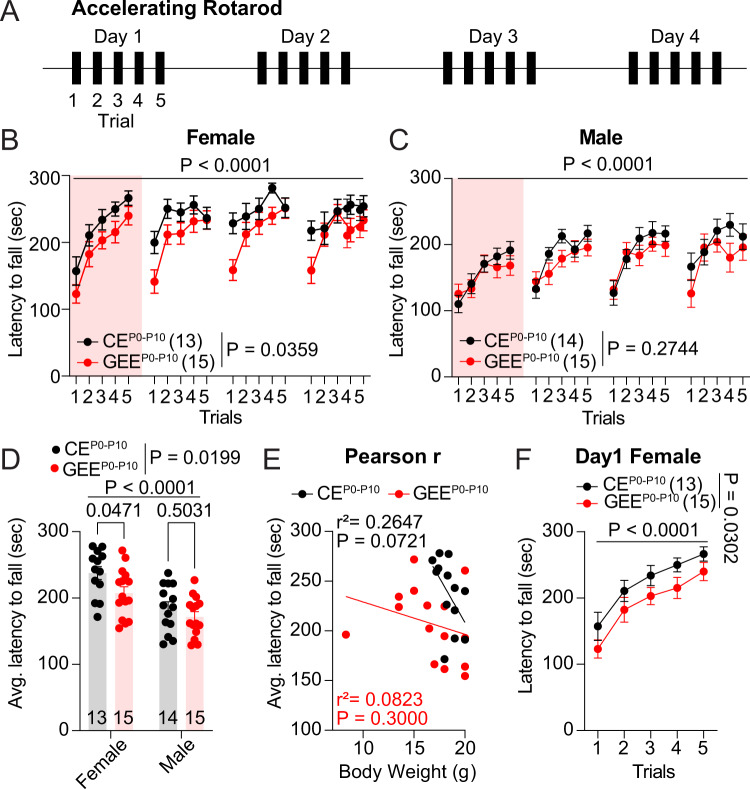
Late-term GEE impairs rotarod performance in adult female offspring. **A** Timeline of behavioral experiments. **B** Latency to fall during rotarod test across trials in CE^P0-P10^ and GEE^P0-P10^ female offspring (RM two-way ANOVA; treatment main effect: F_(1,26)_ = 4.895, *p* = 0.0359; trial main effect F_(9.535,247.9)_ = 11.09, *p* < 0.0001; treatment × trial interaction: F_(19,494)_ = 1.021, *p* = 0.4346). **C** Latency to fall during rotarod test across trials in CE^P0-P10^ and GEE^P0-P10^ male offspring (RM two-way ANOVA; treatment main effect: *F*_(1,27)_ = 1.245, *p* = 0.2744; trial main effect F_(9.198,248.3)_ = 9.598, *p* < 0.0001; treatment × trial interaction: F_(19,513)_ = 0.9056, *p* = 0.5762). **D** Average latency to fall across trials for CE^P0-P10^ and GEE^P0-P10^ female and male offspring (two-way ANOVA; treatment main effect: F_(1,53)_ = 5.766, *p* = 0.0199; sex main effect F_(1,53)_ = 23.74, *p* < 0.0001; treatment × sex interaction: F_(1,53)_ = 0.8473, *p* = 0.3615; followed by Sidak post-hoc test). **E** Pearson correlation between body weight and average performance for CE^P0-P10^ and GEE^P0-P10^ female offspring (CE^P0-P10^: r^2^ = 0.2647, *p* = 0.0721; GEE^P0-P10^: r^2^ = 0.0823, *p* = 0.3000). **F** Latency to fall off the rotarod across trials at day 1 in CE^P0-P10^ and GEE^P0-P10^ female offspring (RM two-way ANOVA; treatment main effect: F_(1,26)_ = 5.255, *p* = 0.0302; trial main effect F_(3.236,84.14)_ = 20.86, *p* < 0.0001; treatment × trial interaction: F_(4,104)_ = 0.0349, *p* = 0.9976). Data are expressed as mean ± SEM. N indicates number of mice.

We used the accelerating rotarod task to test whether GEE^P0-P10^ impairs motor skills (Fig. 2A). We trained adult mice for five trials/session over four consecutive days. One trial lasted a maximum of 300 sec (or until the mouse fell off the rotarod), during which the rod accelerated from 4 to 40 rpm. While GEE^P0-P10^ female offspring displayed motor deficits across trials (Fig. 2B), GEE^P0-P10^ male mice did not (Fig. 2C). Similar to previous experiments [51, 52], the analysis of average performance across trials by sex revealed that CE^P0-P10^ female mice outperformed male CE^P0-P10^ offspring (Fig. 2D). Moreover, the same analysis revealed a decreased latency to fall off the rotarod in GEE^P0-P10^ compared to CE^P0-P10^ female progeny (Fig. 2D). To investigate whether the altered motor skill was due to differences in offspring growth, we performed a correlation analysis between average performance and body weight for each female GEE^P0-P10^ mouse. This analysis did not detect any correlation between the two factors, thus excluding that motor deficits were due to decreased growth in the female offspring (Fig. 2E). Finally, we assessed whether motor impairments were also evident during the first training day in female mice and observed a reduced performance in GEE^P0-P10^ compared to CE^P0-P10^ mice (Fig. 2F). Altogether our data indicate that GEE^P0-P10^ induces sex-specific deficits in motor skills in the female offspring.

### Late-term GEE alters nAChR-mediated regulation of dopamine release in the female offspring

**Fig. 3:**
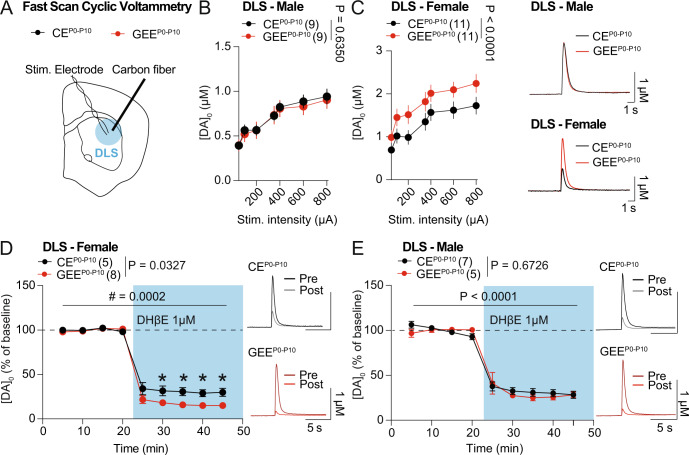
Late-term GEE alters nAChR-mediated regulation of dopamine release in the female offspring. **A** Experimental schematic diagram of Fast Scan Cyclic Voltammetry (FSCV) in DLS of adult mice. **B** Input-output curve of evoked-DA release in DLS of CE^P0-P10^ and GEE^P0-P10^ male offspring (two-way ANOVA; treatment main effect: F_(1,112)_ = 0.2265, *p* = 0.6350; stimulation main effect F_(6,112)_ = 11.48, *p* < 0.0001; treatment × stimulation interaction: F_(6,112)_ = 0.0468, *p* = 0.9996). **C** Input-output curve of evoked-DA release in DLS of CE^P0-P10^ and GEE^P0-P10^ female offspring (two-way ANOVA; treatment main effect: F_(1,140)_ = 18.59, *p* < 0.0001; stimulation main effect F_(6,140)_ =  8.897, *p* < 0.0001; treatment × stimulation interaction: F_(6,140)_ = 0.0807, *p* = 0.9980). **D** Time-course of evoked-DA release in DLS of CE^P0-P10^ and GEE^P0-P10^ female offspring before and after DhβE (1 µM) bath-application (RM two-way ANOVA; treatment main effect: F_(1,11)_ = 5.960, *p* = 0.0327; time main effect F_(8,88)_ = 496.9, *p* < 0.0001; treatment × time interaction: F_(8,88)_ = 4.323, *p* = 0.0002; followed by between-group Sidak post-hoc test, * < 0.05). **E** Time-course of evoked-DA release in DLS of CE^P0-P10^ and GEE^P0-P10^ male offspring before and after DhβE (1 µM) bath-application (RM two-way ANOVA; treatment main effect: F_(1,10)_ = 0.1895, *p* = 0.6726; time main effect F_(3.016,30.16)_ = 178.7, *p* < 0.0001; treatment × time interaction: F_(8,80)_ = 0.9833, *p* = 0.4553).

As dopamine has essential roles in motor skill behavior, we evaluated striatal dopamine release in the DLS of male and female GEE^P0-P10^ mice using FSCV during adulthood (Fig. 3A). We generated an input-output curve by measuring dopamine release evoked by a single electrical pulse stimulation of increasing current intensities. While GEE^P0-P10^ did not affect evoked dopamine release curves in males (Fig. 3B), it increased evoked-dopamine release in female GEE^P0-P10^ offspring compared to CE^P0-P10^ (Fig. 3C). Due to the role of striatal ACh in driving single-pulse evoked dopamine release, we measured the sensitivity of evoked-dopamine release to the nAChR antagonist, DHβE (1 µM), in GEE^P0-P10^ female and male offspring. We observed that GEE^P0-P10^ females had a larger DHβE-induced evoked-dopamine depression compared to CE^P0-P10^ mice (Fig. 3D). These deficits were not detected in the GEE^P0-P10^ male offspring (Fig. 3E). These data indicate that impaired evoked dopamine release might be due to an upregulated function of pre-synaptic nAChRs on dopaminergic terminals.

### Late-term GEE alters striatal acetylcholine dynamics in the female offspring

**Fig. 4:**
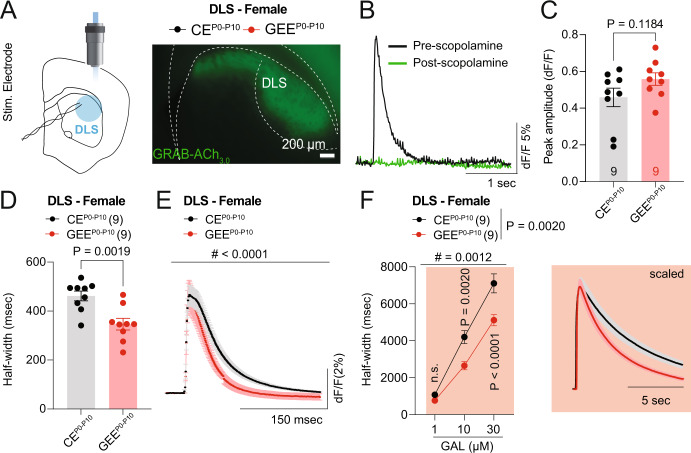
Late-term GEE alters striatal acetylcholine dynamics in female offspring. **A** Experimental schematic diagram of photometry-pharmacology experiments in DLS slices of CE^P0-P10^ and GEE^P0-P10^ female offspring during adulthood. **B** Scopolamine 10 µM blocks dF/F GRAB-ACh_3.0_ transients. **C** Peak amplitude of dF/F transients evoked by 6 pulse stimulation at 100 Hz in DLS of CE^P0-P10^ and GEE^P0-P10^ female mice (unpaired t-test, t_16_ = 1.650). **D** Decay time (half-width) of dF/F transients evoked by 6 pulse stimulation at 100 Hz in DLS of CE^P0-P10^ and GEE^P0-P10^ female mice (unpaired t-test, t_16_ = 3.709). **E** Average dF/F traces evoked by 6 pulse stimulation at 100 Hz from DLS of CE^P0-P10^ and GEE^P0-P10^ female mice (RM two-way ANOVA; treatment main effect: F_(1,14)_ = 4.421, *p* = 0.0541; time main effect: F_(249, 3486)_ = 124.6, *p* < 0.0001; treatment × time interaction: F_(249, 3486)_ = 1.942, *p* < 0.0001). **F** Decay time (half-width) of dF/F transients evoked by 6 pulse stimulation at 100 Hz in DLS of CE^P0-P10^ and GEE^P0-P10^ female mice in presence of 1, 10, and 30 µM galantamine with scaled averaged traces (RM two-way ANOVA; treatment main effect: F_(1,16)_ = 13.61, *p* = 0.0020; drug main effect: F_(2,32)_ = 300.6, *p* < 0.0001; treatment × drug interaction: F_(2,32)_ = 8.403, *p* = 0.0012; followed by between-group Sidak post-hoc test).

To investigate whether striatal dopamine deficits were due to impaired ACh release upon electrical stimulation ex vivo, we utilized the genetically encoded sensor GACh_3.0_ [53] expressed in the DLS of CE^P0-P10^ and GEE^P0-P10^ female adult offspring (Fig. 4A). Electrical stimulation of DLS evoked discrete fluorescent changes measured as dF/F transients and blocked by the mAChR antagonist scopolamine (10 µM), as expected for this muscarinic receptor-based sensor [53] (Fig. 4B). We then monitored the dynamics of ACh transients evoked by brief 6-pulse 100 Hz electrical stimulation at baseline and in the presence of the acetylcholinesterase (AChE) inhibitor, galantamine (10 µM). While we did not observe any difference in the peak amplitude of dF/F transients (Fig. 4C), we found a faster decay of ACh transients in GEE^P0-P10^ compared to CE^P0-P10^ female mice at baseline (Fig. 4D, E). Importantly, a faster decay of ACh transients was still observed in the presence of different galantamine concentrations (Fig. 4F). Altogether these data indicate that GEE^P0-P10^ impairs striatal acetylcholine dynamics, suggesting altered striatal ACh release.

### Late-term GEE decreases striatal CIN excitability in the female offspring

**Fig. 5:**
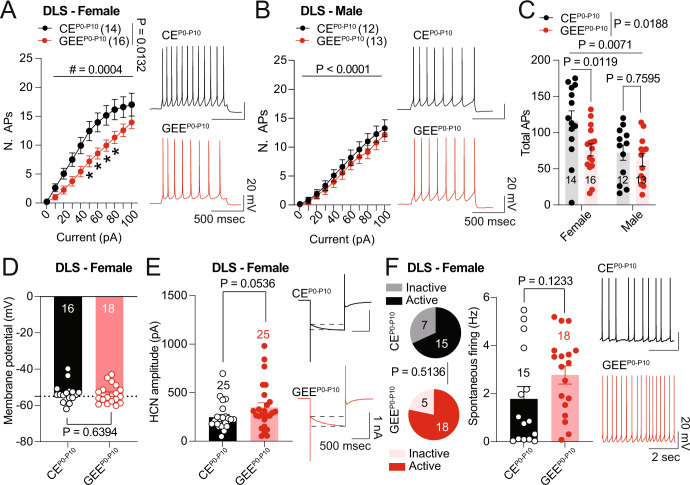
Late-term GEE decreases striatal CIN excitability in the female offspring. **A** Excitability of CINs at increasing positive current steps in DLS of CE^P0-P10^ and GEE^P0-P10^ females (RM two-way ANOVA; treatment main effect: F_(1,28)_ = 7.009, *p* = 0.0132; stimulation main effect F_(10,280)_ = 134.9, *p* < 0.0001; treatment × stimulation interaction: F_(10,280)_ = 3.371, *p* = 0.0004; between-group Sidak post-hoc test, * < 0.05). **B** Excitability of CINs at increasing positive current steps in DLS of CE^P0-P10^ and GEE^P0-P10^ male offspring (RM two-way ANOVA; treatment main effect: F_(1,23)_ = 0.5599, *p* = 0.4619; stimulation main effect F_(10,230)_ = 126.0, *p* < 0.0001; treatment × stimulation interaction: F_(10,230)_ = 0.4667, *p* = 0.9103). **C** Total number of action potentials of CIN in CE^P0-P10^ and GEE^P0-P10^ male and female offspring (two-way ANOVA; treatment main effect: F_(1,51)_ = 5.885, *p* = 0.0188; sex main effect F_(1,51)_ = 7.866, *p* = 0.0071; treatment × sex interaction: F_(1,51)_ = 2.089, *p* = 0.1545). **D** Membrane potential measured in DLS CINs from CE^P0-P10^ and GEE^P0-P10^ female mice (Mann-Whitney U = 130). **E** HCN-mediated current amplitude measured in DLS CINs from CE^P0-P10^ and GEE^P0-P10^ female mice (Mann-Whitney U = 213). **F** Number of active and inactive CINs (Fisher’s Exact Test, *P* = 0.5136) and spontaneous firing rate (Mann-Whitney U = 92) measured in DLS CINs from CE^P0-P10^ and GEE^P0-P10^ female mice. Data are expressed as mean ± SEM. N indicates number of slices and neurons.

To understand whether CIN deficits underlie altered striatal ACh dynamics, we measured the excitability of CINs identified by their large soma size and the presence of a hyperpolarization-activated cyclic nucleotide-gated channel (HCN)-mediated current [54] in both male and female offspring. In female mice, we observed a decreased excitability in GEE^P0-P10^ compared to CE^P0-P10^ CINs (Fig. 5A), which was not detected in male GEE^P0-P10^ offspring (Fig. 5B). The combined analysis of the total number of action potentials also revealed higher CIN excitability in female than male mice, which was decreased by GEE^P0-P10^ (Fig. 5C). We did not observe any difference between the treatment groups in the membrane potential (Fig. 5D), HCN current amplitude (Fig. 5E), number of spontaneously active and inactive CINs, or their spontaneous firing rates (Fig. 5F). These data indicate that the faster decay time of ACh transients might be caused by reduced ACh release associated with a decreased DLS CIN excitability in GEE^P0-P10^ female offspring.

### Cholinergic modulation improves rotarod deficits in late-term GEE offspring

**Fig. 6:**
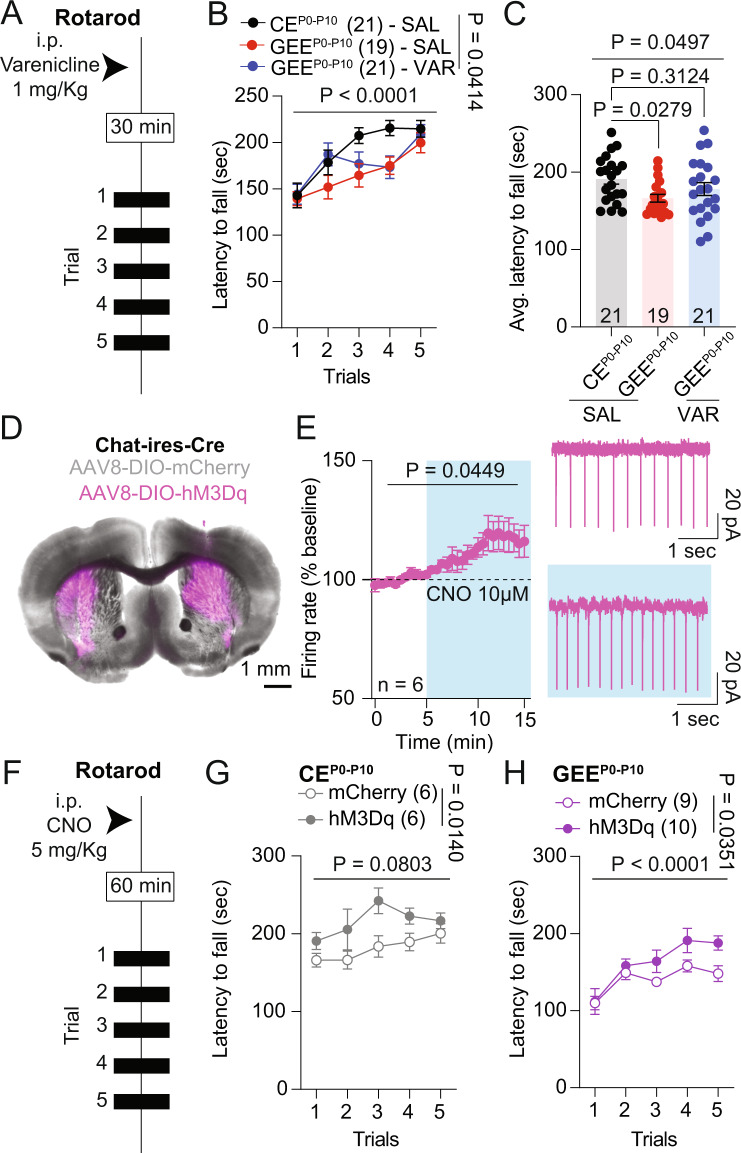
Cholinergic modulation improves motor performance in female mice. **A** Experimental schematic of varenicline treatment and rotarod training in CE^P0-P10^ and GEE^P0-P10^ female offspring. **B** Latency to fall off the rotarod across trials at day 1 in saline (SAL)-treated CE^P0-P10^ and saline (SAL)- or varenicline (VAR)-treated GEE^P0-P10^ female offspring (mixed effects model, REML; treatment main effect: F_(2,58)_ = 3.367, *p* = 0.0414; trial main effect F_(3.739,214)_ = 18.09, *p* < 0.0001; treatment × trial interaction: F_(8,229)_ = 1.607, *p* = 0.1238). **C** Average latency to fall at day 1 trials in CE^P0-P10^ and GEE^P0-P10^ female offspring (one-way ANOVA; F_(2,58)_ = 3.163, p = 0.0497; followed by Dunnet post-hoc test). **D** Representative image of Chat-ires-Cre animals expressing hM3Dq in the dorsal striatum. **E** Normalized firing rate of fluorescently identified CIN expressing hM3Dq before and after CNO bath application (RM ANOVA; F_(1.744,8.718)_ = 4.693, *p* = 0.0449). **F** Experimental schematic of CNO treatment and rotarod training in CE^P0-P10^ and GEE^P0-P10^ female offspring. **G** Latency to fall off the rotarod across trials at day 1 in CNO-treated CE^P0-P10^ female offspring expressing either mCherry or hM3Dq in CINs (mixed effects model, REML; virus main effect: F_(1,10)_ = 8.837, *p* = 0.0140; trial main effect F_(3.048,28.20)_ = 2.485, *p* = 0.0803; virus × trial interaction: F_(4,37)_ = 0.7475, *p* = 0.5660). **H** Latency to fall off the rotarod across trials at day 1 in CNO-treated GEE^P0-P10^ female offspring expressing either mCherry or hM3Dq in CINs (RM two-way ANOVA; virus main effect: F_(1,17)_ = 5.241, *p* = 0.0351; trial main effect F_(2.766,47.03)_ = 11.17, *p* < 0.0001; virus × trial interaction: F_(4,68)_ = 1.145, *p* = 0.3430). Data are expressed as mean ± SEM. N indicates number of neurons and animals.

To link striatal ACh and motor deficits in our late-term GEE model, we first assessed the effects of varenicline, a partial agonist of β2-containing nAChRs [55], on rotarod performance. We systemically administered varenicline (1 mg/Kg) 30 minutes before the beginning of rotarod training sessions in the female CE^P0-P10^ and GEE^P0-P10^ offspring (Fig. 6A). All mice improved their performance across trials, with varenicline administration positively affecting GEE^P0-P10^ performance (Fig. 6B), particularly during the first training trials compared to saline-treated CE^P0-P10^ and GEE^P0-P10^ offspring. The analysis of the average latency to fall off the rotarod revealed that varenicline administration improved GEE^P0-P10^ latency to fall to similar levels of CE^P0-P10^ treated with saline (Fig. 6C).

To assess whether a region and cell-specific rescue of CIN activity improves motor deficits in GEE^P0-P10^ mice, we transduced DLS of CE^P0-P10^ and GEE^P0-P10^ adult female mice with the positive chemogenetic modulator hM3Dq [56] or mCherry control (Fig. 6D). Using brain slice recordings, we observed that bath application of Clozapine-N-Oxide (CNO) increased the firing frequency of CIN expressing hM3Dq (Fig. 6E). Then, we assessed the ability of hM3Dq activation to improve rotarod performance *via* i.p. injections of CNO 60 min before the beginning of rotarod training (Fig. 6F). In CE^P0-P10^, CNO administration improved the performance of hM3Dq-expressing females compared to mCherry-expressing controls (Fig. 6G). CNO also enhanced the performance of GEE^P0-P10^ female mice infected with hM3Dq in DLS compared to mCherry controls (Fig. 6H). Altogether, these data indicate that deficits in DLS CIN function contribute to motor performance impairments, and their activation improves motor deficits in the GEE^P0-P10^ female progeny.

## Discussion

In the present work, we show sex-specific anatomical and striatal circuit deficits in the adult offspring of a late-term GEE mouse model. Adult GEE^P0-P10^ female mice display reduced body weight, increased striatal dopamine release, and impaired nicotinic regulation of electrically evoked dopamine release. These deficits are associated with a reduced decay time of electrically evoked ACh transients, suggesting decreased striatal ACh release, which might be linked to the reduced CIN excitability of GEE^P0-P10^ female offspring. Finally, we show that varenicline treatment and chemogenetic excitation of striatal CINs improve rotarod performance in adult GEE^P0-P10^ female mice. Altogether, our data provide evidence of impaired striatal dopamine and ACh function linked to motor deficits observed in GEE mouse models and patients affected by FASD.

Alcohol exposure during early postnatal days in pups is thought to mimic the effects of ethanol during late gestation in humans [15]. This model is relevant for investigating the adult neurobehavioral sequelae of fetal alcohol exposure due to relapse at later stages of pregnancy [3]. However, some considerations are warranted. First, alcohol metabolism differs between pre and postnatal periods, ultimately impacting circulating ethanol levels. Before birth, the alcohol dehydrogenase (ADH) enzyme expressed in the maternal liver [57] metabolizes alcohol. On the contrary, alcohol metabolism relies on rodents’ low expression of ADH during the first postnatal days [58]. In the GEE^P0-P10^ model, the absence of ADH facilitates reaching binge-like ethanol levels in pups; however, both dams and their progeny are exposed to ethanol vapor. In line with previous studies [49, 50], high alcohol levels impair pup retrieval, a sign of maternal neglect in GEE^P0-P10^. This effect was not observed in an earlier study from the laboratory that utilized lower alcohol level exposures throughout pregnancy and during the first ten postnatal days and in which only male mice were examined [13]. This suggests that impairments in maternal behavior might be dose- and timing-dependent and most likely related to acute maternal intoxication, also depending on the previous history of EtOH exposure and tolerance. Moreover, pup ultrasonic vocalizations are essential contributors to maternal pup retrieval initiation [59] and are affected by ethanol exposure during development [60]. Whether alcohol intoxication might directly interfere with the ability of pups to emit vocalizations and cause maternal neglect in our GEE^P0-P10^ model remains an open question. Further experiments will clarify the factors contributing to impaired maternal care and whether striatal and motor skill deficits derive from an interaction between ethanol exposure and impaired maternal care in the GEE^P0-P10^ offspring.

Our study highlights a greater vulnerability of the female offspring to GEE^P0-P10^ compared to males. Importantly, sex-specific neurobehavioral deficits during adulthood were not associated with significant differences in either alcohol levels in the blood circulation or maternal care between the sexes during infancy. Thus, one hypothesis is that the sex-specific deficits observed in GEE^P0-P10^ female offspring might rely on the combined effects of higher levels of both alcohol and estrogens. Similar to previous evidence [61], CE^P0-P10^ female mice displayed higher dopamine levels than the control male progeny. These effects are thought to be mediated by baseline differences in dopamine release [62]. Moreover, fluctuations in ovarian hormones interact with nAChR and dopamine receptor 2 (D2R) to promote and reduce dopamine release *via* dopamine-mediated autoinhibition [62], respectively. Despite higher dopamine levels in CE^P0-P10^ female mice, we observed a further increase in the female GEE^P0-P10^ progeny, which suggests an interaction between sex and prenatal alcohol exposure. This idea is supported by previous evidence indicating that estradiol increases dopamine neuron sensitivity to ethanol in adult mice [63]. Future research will investigate whether decreasing estrogen levels in GEE^P0-P10^ female offspring through hormonal therapy or ovariectomy will rescue the neurobehavioral deficits observed in the adult offspring. In addition to the alcohol-estradiol interaction hypothesis, male and female offspring might adopt distinct behavioral strategies by recruiting different brain pathways during action execution [64, 65]. These circuits might be differentially affected by ethanol exposure. In this framework, future studies will investigate whether female mice would rely on CIN activity during motor skill learning, which is affected by GEE^P0-P10^, and might justify the observed sex-specific behavioral effects.

Our electrophysiology, voltammetry, and photometry experiments contribute to a circuit model of GEE^P0-P10^-induced striatal adaptations whereby decreased striatal CIN excitability impairs ACh release, motor behavior, and promotes a compensatory upregulation of nAChRs to drive higher electrically evoked dopamine release. Based on our data, a potential driver for ACh release deficits could be the reduced DLS CIN excitability observed in GEE^P0-P10^ adult female progeny. This effect might be sex-, region-, and cell-specific, considering that previous authors observed a decrease in MSN excitability in the posterior portion of the dorsomedial striatum (DMS) in GEE adult male progeny [66]. However, whether GEE^P0-P10^ induces similar deficits in other striatal subdivisions in the male progeny remains an open question.

The absence of changes in basal membrane properties of DLS CINs opens the possibility that GEE^P0-P10^ might affect intra-striatal and striatal afferent function to impair CIN intrinsic excitability. For example, GEE decreases inhibitory transmission on MSNs while increasing their excitability [13]. Moreover, fetal alcohol exposure alters excitatory transmission in several brain areas projecting to the striatum [16, 19], including the prefrontal cortex [67, 68] and the somatosensory cortex [69]. Within the striatum, GEE alters excitatory synaptic function on MSNs in DMS [70] and DLS [32]. Along with deficits in basal glutamatergic transmission, GEE impairs high-frequency stimulation (HFS)-induced long-term depression (LTD) of excitatory synapses on MSNs, which is recovered by the bath application of a D2R agonist [71]. In striatal CINs, HFS also induces a form of N-methyl-D-aspartate receptor (NMDAR)- and dopamine receptor 1 (D1R)-dependent synaptic potentiation [72]. Thus, one additional mechanism that may account for decreased CIN excitability deficits might be a GEE^P0-P10^-mediated alteration in basal synaptic transmission and plasticity.

Dopamine modulates CIN activity, effectively establishing a striatal acetylcholine-dopamine balance essential for decision-making and action execution [73]. While ACh regulates dopamine release *via* β2-containing nAChRs during single-pulse stimulation [27–30], D2R activation controls CIN firing [74] and ACh release in ex vivo [75, 76] and in vivo [22, 23] striatal preparations. Of note, GEE alters the function of the dopamine transporter, D1R, and D2R in the striatum of rats [31] and monkeys [33]. Thus, these data and our observations of a GEE^P0-P10^-mediated increase in single-pulse evoked dopamine release highlights the possibility that dopamine deficits converge to alter synaptic plasticity and CIN excitability in the female progeny.

Our photometry data show that GEE^P0-P10^ does not affect the peak amplitude of ACh transients but decreases their decay time, suggesting a higher rate of ACh degradation by the AChE. To assess whether blockade of AChE would rescue the heightened decay time of ACh transients in GEE^P0-P10^ female mice, we bath-applied the AChE inhibitor galantamine while measuring stimulation-induced ACh release. Previous experiments [77] in human and rat cortical homogenates indicate an almost complete inhibition of AChE activity at the range of 10-30 µM galantamine doses used in our study. The persistence of a faster decay in the presence of 10–30 µM galantamine suggests that reduced release, rather than heightened AChE activity, underlies faster evoked ACh transients in GEE^P0-P10^ female mice. Our data corroborate previous evidence that postnatal ethanol exposure does not alter AChE levels in the NBM, cortical, and striatal regions [35, 36]. Moreover, other experiments demonstrated that GEE, particularly during prenatal periods, induces a decrease -rather than an increase- in AChE expression [78]. While the lower CIN excitability supports our hypothesis of decreased striatal ACh release in the DLS of GEE^P0-P10^ female offspring, future studies will investigate whether GEE also dysregulates ACh synthesis [34], vesicular ACh packaging [76], mAChR- [79, 80] and D2R-mediated inhibition of ACh release [22, 23, 75, 76].

In our model of GEE^P0-P10^, we observed an increased electrically evoked striatal dopamine release associated with increased sensitivity to the nAChR antagonist DhβE during single-pulse electrical stimulation. One hypothesis is that GEE^P0-P10^ upregulates nAChRs to increase single-pulse evoked DA release. To our knowledge, no previous studies show a GEE-induced increase in nAChR function on striatal dopamine terminals; however, alcohol potentiates nAChR-mediated responses [81], and nAChR antagonist blocks some of its behavioral effects in rodents [82]. Moreover, fetal nicotine exposure due to tobacco use alters nAChR subunit composition [83] and upregulates their expression [84, 85]. Thus, GEE might induce persistent upregulation in ACh signaling on DA terminals that depends, at least in part, on the direct effects of alcohol on nAChRs [86, 87] in the developing brain. Future experiments will explore the mechanisms of GEE-induced changes in the expression or subunit composition of pre-synaptic nAChRs on dopamine terminals.

To challenge our GEE^P0-P10^ circuit model, we have conducted pharmacological and chemogenetic experiments to manipulate the activity of nAChRs and CINs, respectively, during rotarod training. In the first case, we administered varenicline, a partial agonist of β2-containing nAChRs. Previous studies have shown that varenicline administration improves motor deficits [88] and alcohol-induced learning defects in rodents [89]. We observed that varenicline administration ameliorated the motor skill deficits in the average performance of female GEE^P0-P10^ offspring, highlighting the contribution of nAChR dysfunctions to these behavioral impairments. Based on previous evidence of CIN activation-mediated improvement of motor function [90], we assessed whether the acute chemogenetic-mediated increase in CIN activity would improve motor skills in our model. We observed that hM3Dq activation increased the latency to fall in both CE^P0-P10^ and GEE^P0-P10^ female offspring treated with CNO. These data provide a circuit-based mechanism supporting previous evidence that dietary supplementation with choline, an ACh precursor, ameliorates spatial learning and cognitive deficits in animal models of fetal alcohol exposure [91, 92].

Altogether this set of experiments highlights novel sex-specific mechanisms of striatal dysfunctions in a mouse model of fetal alcohol exposure. Our findings contribute to the identification of novel therapeutic targets to treat the behavioral symptoms observed in FASD patients.
